# Recent Developments in Vaccine Design: From Live Vaccines to Recombinant Toxin Vaccines

**DOI:** 10.3390/toxins15090563

**Published:** 2023-09-08

**Authors:** Sonal Gupta, Sabine Pellett

**Affiliations:** Department of Bacteriology, University of Wisconsin-Madison, Madison, WI 53706, USA; sgupta279@wisc.edu

**Keywords:** recombinant vaccines, vaccine, toxin, toxoid, botulism, tetanus, COVID-19, mRNA vaccines

## Abstract

Vaccines are one of the most effective strategies to prevent pathogen-induced illness in humans. The earliest vaccines were based on live inoculations with low doses of live or related pathogens, which carried a relatively high risk of developing the disease they were meant to prevent. The introduction of attenuated and killed pathogens as vaccines dramatically reduced these risks; however, attenuated live vaccines still carry a risk of reversion to a pathogenic strain capable of causing disease. This risk is completely eliminated with recombinant protein or subunit vaccines, which are atoxic and non-infectious. However, these vaccines require adjuvants and often significant optimization to induce robust T-cell responses and long-lasting immune memory. Some pathogens produce protein toxins that cause or contribute to disease. To protect against the effects of such toxins, chemically inactivated toxoid vaccines have been found to be effective. Toxoid vaccines are successfully used today at a global scale to protect against tetanus and diphtheria. Recent developments for toxoid vaccines are investigating the possibilities of utilizing recombinant protein toxins mutated to eliminate biologic activity instead of chemically inactivated toxins. Finally, one of the most contemporary approaches toward vaccine design utilizes messenger RNA (mRNA) as a vaccine candidate. This approach was used globally to protect against coronavirus disease during the COVID-19 pandemic that began in 2019, due to its advantages of quick production and scale-up, and effectiveness in eliciting a neutralizing antibody response. Nonetheless, mRNA vaccines require specialized storage and transport conditions, posing challenges for low- and middle-income countries. Among multiple available technologies for vaccine design and formulation, which technology is most appropriate? This review focuses on the considerable developments that have been made in utilizing diverse vaccine technologies with a focus on vaccines targeting bacterial toxins. We describe how advancements in vaccine technology, combined with a deeper understanding of pathogen–host interactions, offer exciting and promising avenues for the development of new and improved vaccines.

## 1. Introduction

One of the most effective methods of preventing diseases in humans and animals is the population-wide use of vaccines [1,2]. A vaccine is a biological formulation designed to give rise to a protective and ideally long-lasting immune response against a pathogen without causing disease. Many different approaches have been employed in developing vaccines. Most commonly, vaccines consist of a killed or attenuated form of the entire pathogen, or a surface protein or toxin derivative. All vaccines prime and stimulate the host’s immune system to recognize the pathogen as a threat and rapidly eliminate it from the system, hence, protecting the host from severe disease after infection with a pathogen or after intoxication. Vaccines can be prophylactic or therapeutic, where prophylactic vaccines are given prior to encounter of the pathogen, and therapeutic vaccines are administered after encounter of the pathogen [1,2].

The history of vaccines can be traced back to the late 18th century when smallpox was rampant and caused many deaths across Europe. After observations that smallpox survivors were subsequently immune to the disease, human inoculations with a lancet dipped into a ripe smallpox pustule (variolation) became commonplace. While this practice was protective, side effects were serious and frequent, with inoculated persons usually developing mild disease and 2–3% dying from smallpox [3]. In addition, variolated people sometimes caused new outbreaks of smallpox. Towards the end of the 18th century, Edward Jenner, who himself received variolation at the age of 8, noticed that milkmaids who had contracted cowpox, a similar but milder disease, did not get smallpox [4,5]. In an effort to reduce the side effects of variolation, Jenner in 1796 conducted an experiment in which he inoculated a young boy with a fresh cowpox pustule and later exposed him to smallpox. The boy did not get sick, proving that cowpox provided immunity against smallpox. Jenner called this procedure vaccination, based on the Latin word vacca for cow, and vaccinia for cowpox. It took almost 45 years before vaccination eventually replaced variolation in 1840, and after multiple rounds of vaccine alterations to improve safety, the smallpox vaccine eventually developed into one of the greatest medical success stories of humankind, when the disease was eradicated globally in 1977 [3]. Following the initial success of the smallpox vaccine, other vaccines have been developed for a wide range of diseases including measles, mumps, rubella, polio, tetanus, diphtheria, pertussis, hepatitis B, and human papillomavirus (HPV), among others. Emil von Behring and Shibasaburo Kitasato developed the diphtheria antitoxin in the 1890s [4]. Vaccines for pertussis (whooping cough) and tuberculosis were developed in the 1920s [4,5]. Jonas Salk developed the first polio vaccine in the 1940s, which was later replaced by Albert Sabin’s oral polio vaccine. Later, in the 1950s, vaccines for measles, mumps, and rubella were developed [4,5]. The first vaccines for hepatitis B were developed in the 1960s. These developments of vaccines has greatly reduced the incidence and severity of human diseases and has saved countless lives.

**Figure 1:**
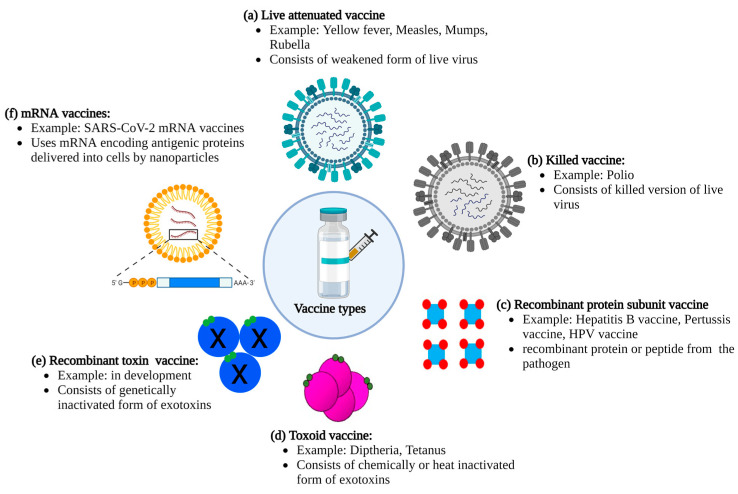
Description of different types of vaccine: (**a**) live attenuated vaccine; (**b**) killed vaccine; (**c**) recombinant protein subunit vaccine; (**d**) toxoid vaccine; (**e**) recombinant toxin vaccine; (**f**) mRNA vaccine. Image created with BioRender.com (accessed on 1 August 2023).

The smallpox vaccine was developed without an understanding of the virus underlying the disease. Currently, our detailed organismal, molecular, and structural insights into various pathogens enable highly sophisticated and targeted approaches in vaccine developments, dramatically improving the safety of vaccines. Based on the pathogen against which a vaccine is designed (bacteria or virus), there are multiple vaccine technologies to generate a protective immune response (Figure 1). Live attenuated vaccines consist of live pathogens from either a bacterium or a virus that has been weakened through heat or chemical methods. These vaccines contain live pathogens that produce a mild infection but do not result in severe disease, thereby inducing protective cells mediated as well as humoral immunity with strong, effective, and long-lasting protection after only one inoculation [6]. However, one of the major drawbacks of live attenuated vaccines is the risks associated with these kinds of vaccines, including a risk of reversion of the vaccine strain to a virulent strain, leading to disease. In addition, certain populations such as immunocompromised people may be more susceptible to developing disease from an attenuated pathogen. Killed vaccines overcome this issue by not containing any live pathogens. These vaccines, however, still contain the entire pathogen, thus displaying the same immune epitopes capable of initiating a robust immune response. However, killed vaccines usually are less effective than live vaccines in inducing long-lasting protective immunity [6] and require booster shots along with adjuvants to stimulate a strong immune response. Both attenuated and killed vaccines are comparatively inexpensive to make and can be produced on a mass level. 

With the emergence of genetic engineering technology in the late 20th century, approaches towards vaccine formulation included the development of recombinant protein vaccines [7]. Recombinant subunit vaccines consist of recombinantly produced subunits of a pathogen that stimulates the host’s immune system, such as surface proteins or polysaccharides, along with effective adjuvants and, if needed, conjugate carriers [8,9,10]. An important advantage of this technology is that these vaccines consist of only a part of the pathogen rather than the whole organism, and thus they cannot contain or revert to a live pathogen. The first recombinant subunit vaccine was produced in the mid-1980s against hepatitis B, followed by developments of vaccines against human papillomavirus (HPV), flu, herpes zoster, and coronavirus [9,11,12]. Some antigens of pathogens, including polysaccharides and small peptides, were found to be weak in eliciting a protective immune response due to small size or other structural constraints. Such antigens benefit from formulation as conjugate subunit vaccines, integrating a weak antigen with a strong antigen such as a large protein, as a carrier. Recombinant subunit vaccines are restricted in that they elicit an immune response only against the provided antigen, not the entire pathogen. This can potentially result in weakening protection against pathogens that develop mutations in the protein used as an antigen, and a need for more frequent developments of updated vaccines. While these vaccines generally have improved safety profiles compared to whole pathogen vaccines, the antigens themselves theoretically could retain a potential to cause adverse effects.

A similar strategy used for recombinant subunit vaccines is also applicable to vaccines targeting protein toxins. Some pathogens produce exotoxins that contribute to or cause disease, such as tetanus toxin, botulinum toxin, and diphtheria toxin [13]. The toxin’s pathogenic effects can be lessened in severity by immunization with toxoid vaccines, which mount a protective immune response. Toxoid vaccines consist of chemically inactivated toxin mixed with an adjuvant [14]. Multiple boosters are usually required to achieve moderately long-term protection. Newer developments utilize structure–function and pathogenicity knowledge to create non-toxic sub-domains or genetically inactivated toxins as vaccines to increase safety during production and decrease side effects related to residual formalin used in toxoid preparation [15,16,17].

One of the most recent vaccine strategies that has been in development for the past 3 decades uses deoxyribonucleic acid (DNA) or messenger ribonucleic acid (mRNA) encoding antigens as vaccine candidates [18,19]. This strategy builds on the protein subunit vaccines, but instead of producing recombinant proteins followed by purification and formulation with adjuvants, nucleic acid vaccines use a carrier to deliver DNA or mRNA encoding the proteins to host cells, where the proteins are then produced by the host and released into the circulation to elicit an immune response. The mRNA vaccine strategy received a huge boost in 2020 when US and European governments supported developments of mRNA-based coronavirus disease (COVID) vaccines in an effort to quickly produce enough vaccine doses to immunize large parts of the population in an effort to combat the COVID pandemic.

With so many different vaccine designs, it is tempting to make claims about one technology being better than another. However, many factors need to be considered in choosing the best vaccine approach for each pathogen. Irrespective of vaccine design and formulation, much research and progress remains to be made in the vaccine field, and there’s a constant need to develop new vaccines and vaccine approaches. To date, there are no protective vaccines available against fatal diseases such as malaria, shigella, dengue, cholera, and paratyphoid fever. Other common diseases, such as flu and coronavirus, require new developments each year to protect against mutating viruses. This review will discuss various vaccine approaches with a focus on protein vaccines, using prominent examples such as Tetanus, Botulism, and Coronavirus Disease 2019 (COVID-19) to highlight the advantages and disadvantages of various approaches.

## 2. Live Attenuated Vaccines

Since live pathogen inoculation was banned in 1840, live attenuated or inactivated pathogens were the first vaccines developed and widely distributed. These vaccines proved to be one of the most cost-effective and successful methods to prevent human disease, and the approach continues to be used today, including the mumps, measles, and rubella (MMR) vaccine and the chickenpox vaccine. Live attenuated vaccines are known to elicit a broad immune response with efficient and rapid production of protective antibodies produced by B cells as well as cellular CD4+ and CD8+ T lymphocyte (T-cells) responses. This robust immune response usually leads to long-lasting protection and immune memory, potentially life-long, with only one or two administrations. Thus, there often is no requirement for multiple booster administration [20]. The immune response generated by live attenuated vaccines mimics the immune response to live pathogen infection. This includes the production of antibodies by B cells as well as the activation of a cellular immune response involving T cells. Both B-cells and T-cells can differentiate into memory cells, which “remember” the pathogen and allow for a quicker and more effective response in the event of future exposure. Development of B cell memory (long-lived plasma cells or memory B cells) proceeds via two consecutive phases, with both T cells and antigen complexity playing an important role in guiding the development of long-lived plasma cells and memory B cells from naïve B cells [21]. As lifelong protection against disease is the most desirable outcome of vaccination, live attenuated vaccines are an excellent choice for some pathogens. However, there are also several drawbacks to these vaccines. When injected, the attenuated pathogen can theoretically revert to a pathogenic strain at a very low frequency, although this has only been observed for the polio vaccine [22]. Since these vaccines will cause an infection, though much milder than the active pathogen, systemic adverse reactions such as fever, headache, rash, myalgia, etc., are commonly experienced by people after immunization. Side effects may be more severe in immunocompromised or chronically ill [22] individuals. The vaccines also need to be kept cold, thus, making travel and global distribution challenging. The MMR vaccine is one of the most widely used live attenuated vaccines today. Originally created as three distinct vaccines, weakened strains of measles, mumps, and rubella were successfully combined into a single vaccine in 1971 [23]. The MMR vaccine and a quadrivalent vaccine, which includes live attenuated varicella virus, are highly effective as demonstrated by high rates of seroconversion in vaccinated populations (measles: 97.1%, mumps: 96.0%, rubella: 98.8%, and varicella: 93.5%) [24]. In the case of the MMR vaccine, the incidence of serious side effects is about 0.03–0.005% according to the Centers for Disease Control (CDC) and a large study in Finland [25].

## 3. Killed Vaccines

After the huge success of live attenuated vaccines, which eradicated one of the worst infectious diseases of humanity, smallpox, there was an attempt to develop equally cost-efficient and effective vaccines with a greater safety profile, by completely killing the pathogen. Killed vaccines are unable to reproduce inside the host and, as a result, cannot spread, revert to a harmful state, or transmit the disease to others [26]. However, the killed pathogens in killed vaccines proved less effective at inducing a protective immune response and memory, often necessitating formulation with adjuvant systems to increase immunogenicity, and multiple doses to achieve long-lasting protection [26]. Some examples of killed vaccines include the polio vaccine [27] (inactivated poliovirus vaccine, IPV) and the rabies vaccine (inactivated rabies virus vaccine) [27,28]. These vaccines are produced by completely killing the infectious agent through various methods such as heat, chemicals, or radiation, which renders them unable to replicate and cause disease [26]. Inactivated vaccines improved the safety profile of vaccines. For example, the attenuated (oral) Polio vaccine first developed by Sabin in 1961 (OPV) resulted in virus reversion and vaccine-induced paralytic poliomyelitis in 1 in 1.4–3.4 million first vaccine doses administered [29]. To avoid these serious adverse events, the OPV vaccine was replaced with the inactivated polio vaccine developed by Salk in 1955 (IPV) in many countries, and no serious adverse events have been attributed to the IPV vaccine. However, the IPV vaccine also has several disadvantages. It costs five times as much to produce, and it primarily induces a humoral immune response and requires boosts to prime the immune system for memory [30]. More importantly, it induces a much lower mucosal immune response in the intestinal tract, which allows the polio virus to infect the intestinal tract of vaccinated individuals without causing systemic disease but allowing the virus to be shed in their feces, thereby contributing to virus spread during outbreaks.

Finally, manufacturing of killed vaccines involves large-scale in vitro culturing of the causative microorganism. Stringent regulatory requirements make it increasingly challenging to gain acceptance for the development of new vaccines of this type for human use [31]. In addition, one of the challenges of producing inactivated vaccines is to ensure effective inactivation while also preserving protective antigens [26]. In spite of the success of the inactivated polio vaccine, severe adverse events including allergic reactions to vaccine components such as vaccine antigen, preservatives, stabilizer or residual animal protein, and neurologic reactions remain a concern for inactivated vaccines. For the rabies vaccine, severe allergic reactions are observed in 3–6% of patients given the entire three-boost schedule, and 6 cases of neurologic adverse events have been reported including 5 cases of Guillaine–Barre syndrome, according to the CDC [32]. 

## 4. Toxoid Vaccine

**Figure 2:**
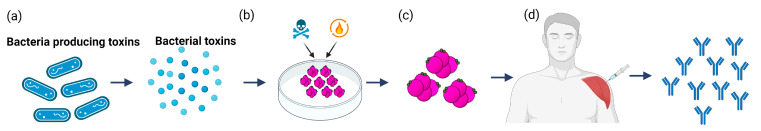
Schematic representation to understand the formulation and working of toxoid vaccines: (**a**) The pathogenic bacteria secrete toxins (exotoxins) that contribute to disease, (**b**) Toxins are inactivated or attenuated using heat or formalin resulting in creation of toxoids, (**c**) The toxoids are absorbed with adjuvants to increase immune response, and (**d**) The host body generates antibodies in response to toxoid, providing immunity against the toxin. Image created with BioRender.com (accessed on 1 August 2023).

Several human pathogens produce exotoxins, and vaccines against the exotoxins have proven invaluable for public health [4]. Toxoid vaccines have been used since the 1920s and are known to elicit a strong and protective humoral immune response after initial vaccination [33,34], but several booster shots are required to develop longer-term immunity [33,34]. Unlike a vaccine against an entire organism or virus particle, which contains multiple immunogenic epitopes including antigens derived from surface protein epitopes, viral capsids, bacterial cell walls, glyco-epitopes, and polysaccharide epitopes, a toxoid vaccine contains a single protein. However, within the single protein, multiple antigenic epitopes are present, some of which are more immunogenic than others. The process of turning a biologically active toxin into a toxoid usually involves prolonged chemical treatment with formalin to inactivate the toxin. When the toxoid vaccine is injected into the body, it is recognized as foreign and results in a strong humoral immune response, producing antibodies directed against specific antigenic epitopes preserved on the toxoid structure. With repeated boosts, the immune system develops a moderate humoral memory response lasting for several years, allowing for a rapid and robust response with future exposure. However, repeated boosts are required periodically to maintain lifetime protection. Toxoid vaccines can also, to a lesser extent, stimulate a T cell response contributing to protection against the disease [35]. When a toxoid vaccine is administered, the antigen-presenting cells (APCs) such as dendritic cells generally process the toxoid and present peptide antigens on their cell surface using major histocompatibility complex class II (MHC-II) molecules. These MHC-II-peptide complexes act as a signal for T cells, specifically CD4+ T cells (helper T cells) and trigger a cascade of immune responses, resulting in the activation of B cells and cytokine secretion in the host body, ultimately leading to differentiation of B cells to antibody-producing plasma cells and memory B cells [36] (Figure 2). Prime examples of toxoid vaccines are tetanus and diphtheria vaccines, which use chemically inactivated toxoid combined with an adjuvant such as Alum [37] to effectively protect against the disease and will be discussed in more detail below. 

### 4.1. Tetanus Toxoid Vaccines

**Figure 3:**
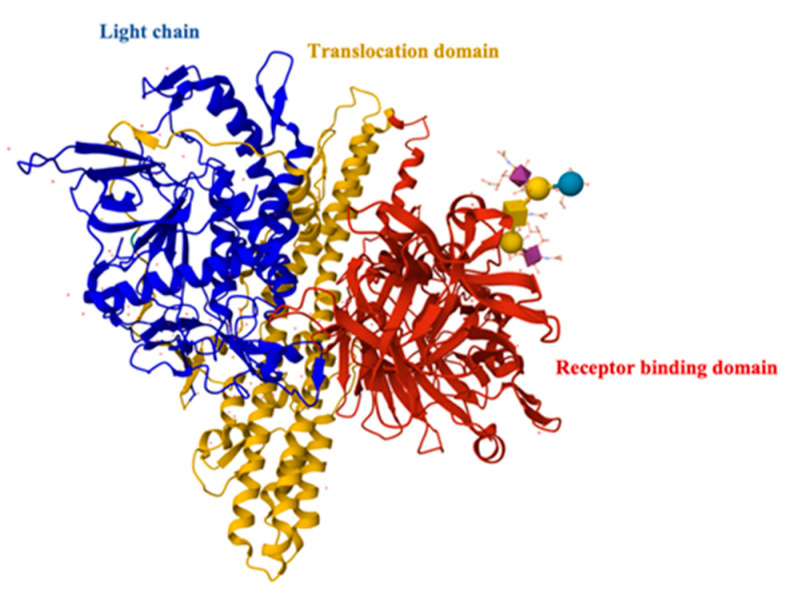
Crystal structure of the tetanus neurotoxin (5N0B): crystal structure of the tetanus neurotoxin in complex with GD1a. The image was obtained from the Protein Data Bank (PDB), ID 10.2210/pdb5N0B/pdb [40].

Tetanus, a neuromuscular syndrome, is caused by the tetanus neurotoxin produced by the anaerobic, Gram-positive, spore-forming bacterium *Clostridium tetani* after deep wound infection [38,39,40]. Clostridium tetani produces tetanus toxin (TeNT) as a single-chain 150 kDa toxin that is proteolytically converted to the active di-chain toxin consisting of N-terminal protease light chain (LC) linked by a disulfide bond to C-terminal heavy chain (HC). The HC further consists of a receptor-binding domain (H_C_) and a LC translocation domain (H_N_) [41] (Figure 3). TeNT specifically binds to host neurons via polysialoganglioside (GT1b, GD1b) interaction, leading to endocytosis of the entire toxin. The toxin then undergoes retrograde and anterograde transport to the central nervous system, where it enters inhibitory interneurons via transcytosis [42]. In the inhibitory neurons, the LC is released into the cell cytosol via H_N_-mediated translocation. The LC then cleaves neuronal vesicle-associated membrane protein 2 (VAMP2), which is an essential part of the exocytosis machinery of neurons, and thereby inactivates neurotransmission from inhibitory neurons. This results in over-firing of the activating neurons and associated spastic paralysis [42].

*C. tetani* spores are found in soil, dust, manure, and saliva. Exposure of wounds, deep cuts, or burns to the spores can result in germination and vegetative growth of the bacteria in the anaerobic environment of the deep wound, accompanied by TeNT production, which spreads throughout the body via the circulatory system [38,39,40]. Tetanus once was a major global disease and today remains a significant threat in many parts of the world, specifically in low-income nations because of lower immunization coverage and unsafe or unhygienic birth practices [36,37,38]. In 2015, the Global Burden of Disease Study indicated that around 56,000 deaths per year were caused by tetanus worldwide. Treatment for this disease involves administering supportive care, passive immunization with tetanus immunoglobulin, and eradicating bacteria at the site of the wound. Once tetanus develops, the disease causes extreme pain and muscle spasms so strong that they may result in the breakage of bones. The case fatality rate of tetanus remains high (up to 85%), even in areas with abundant medical resources. However, currently, TeNT toxoid vaccines approved by the World Health Organization are included in regular immunization schedules on a global level, dramatically reducing the incidence of tetanus. The TeNT vaccine was first developed in 1924 and is now part of the recommended immunization schedule for children, as it has proven to be effective in preventing tetanus. As of 2018, approximately 85% of infants worldwide (116.3 million infants) had received three doses of the combined diphtheria–tetanus–pertussis vaccine.

The tetanus–toxoid vaccine is based on formalin-inactivated full-length tetanus toxin. Although efficient, these vaccines require complex manufacturing procedures along with pollution and production risks associated with formaldehyde use [43,44]. In addition, large batches (typically 1000 L) of *C. tetani* strain Harvard are fermented for 4–7 days, allowing for the vegetative growth of the bacteria accompanied by active toxin production. The active toxin is then isolated from the bacteria and precipitated by multiple biochemical steps. The yield of semi-purified active TeNT per liter of culture is around 1–10 mg/L, meaning vaccine producers handle extremely large amounts of this potentially lethal and potent toxin, which has an estimated human lethal dose of 0.2 ng/kg intramuscularly [45]. The semi-purified active TeNT is then subjected to a formaldehyde inactivation step, which involves incubation with 0.2–1% formaldehyde for a period of around 6 weeks. This inactivation step results in a completely inactivated toxoid, which can be used for vaccination [46]. While formaldehyde inactivation of protein toxins sounds simple, studies have shown complex chemical modifications resulting in a heterogeneous mixture of adducts and cross-links, which can alter the displayed epitopes and antigen processing [47,48,49]. Therefore, there is a crucial need for good batch-to-batch consistency, as variation in the antigen structure due to detoxification may result in different efficiency of the final vaccine batch [47]. While formaldehyde treatment generally increases the immunogenicity and stability of the respective protein overall, the extensive chemical alterations occurring to the proteins combined with potentially increased susceptibility to proteolytic degradation [50] potentially result in changes in immunogenic epitopes. It is currently unknown whether such changes may affect the efficacy of the vaccine. Thus, it remains to be elucidated whether formaldehyde treatment is beneficial or detrimental to the immunogenicity of specific protein toxins.

TeNT toxoid vaccines usually are combined with other vaccines, such as the DTaP vaccines (diphtheria, tetanus, and pertussis), Td vaccines (tetanus and diphtheria), and DT vaccines (diphtheria and tetanus). The diphtheria, pertussis and tetanus (DPT) vaccine is a trivalent vaccine consisting of DT toxoid, TeNT toxoid, and either killed whole cells of *Bordetella pertussis* or pertussis antigen [51]. The CDC (Centre for Disease Control) suggests completion of a series of DTaP or DT between ages 6 weeks and 6 years (at 2 months, 4 months, 6 months, between 15 and 18 months, and between 4 and 6 years of age), a dose of Tdap or Td at age 11 to 12 years, and subsequent boosters every 10 years. There is a need for regular boosters of the TeNT vaccine about every 10 years due to the vaccine eliciting only a moderate memory response [52]. As a result, the TeNT vaccines are often unsuccessful in low-income and developing nations, which already suffer from low vaccination coverage, due to an unwillingness to receive multiple doses and boosters [53,54]. Thus, there is interest in the development of a new vaccine against tetanus neurotoxin, ideally with lifelong protection from one vaccination schedule.

### 4.2. Diphtheria Toxoid Vaccines

Diphtheria, a severe respiratory infection, is primarily caused by toxigenic strains of the Gram-positive, invasive bacterium *Corynebacterium diphtheria* [55]. While *C. diphtheriae* is an invasive bacteria that enters respiratory tissue host cells via adhesins, haemagglutinins, and surface-exposed non-fimbrial proteins, which contributes to pathogenicity, a major virulence factor causing host cell death is the secreted diphtheria exotoxin (DT). DT is an elongation factor 2 (EF2) adenosine diphosphate (ADP) ribosylating toxin [56]. DT is a 58 kDa polypeptide that is secreted across the cytoplasmic membrane of *C. diphtheria* without cell lysis [55]. Like CNTs, DT is a disulfide-linked dichain protein consisting of the catalytic (C) subdomain and a carboxyl-terminal subdomain containing the translocation (T) and receptor-binding (R) domains. The R domain of DT binds heparin-binding epidermal growth factor (HB-EGF) receptors on human epithelial cells, leading to clathrin-mediated endocytosis of the entire toxin-receptor complex. The acidic conditions in the endosome trigger a conformational change leading to membrane insertion of the T domain and translocation of the C domain into the cell cytosol, where it catalyzes the transfer of ADP from nicotinamide adenine dinucleotide (NAD) to EF-2 [55]. Since EF-2 is an essential factor for cell protein biosynthesis, ADP ribosylation by DT prevents proper EF-2 function, thereby shutting down host cell protein synthesis ultimately leading to cell death [55].

It is estimated that before the 1980s, around one million diphtheria cases occurred annually on a global level. The diphtheria toxoid vaccine, which is prepared similarly to tetanus toxoid by inactivating the toxin with formalin, was first developed in 1920 and was successful in preventing serious disease. Vaccine use decreased global diphtheria cases to ~100,000 by 1980, when more widespread use of the vaccine resulted in a strong and steady decline in case numbers [55]. In 2015, 4500 cases were officially reported, a notable decrease from the nearly 100,000 cases recorded in 1980 according to WHO reports [57]. Presently, diphtheria is most prevalent in sub-Saharan Africa, India, and Indonesia. Although rare in developed countries due to widespread vaccination, there is a risk of resurgence if vaccination rates decline. Between 1980 and 2004, the United States reported only 57 cases.

The immune response stimulated by diphtheria toxoid is similar to that stimulated by tetanus toxoid. Like tetanus toxoid, diphtheria toxoid vaccines are relatively cost-effective to produce, but have several downsides including the need for the production of a potent and dangerous toxin at a large scale, differences in the purity of the protein toxin due to varying purification processes, the long chemical inactivation procedure leading to batch to batch differences, and the need to use formalin.

### 4.3. Botulinum Neurotoxin Toxoid Vaccines

**Figure 4:**
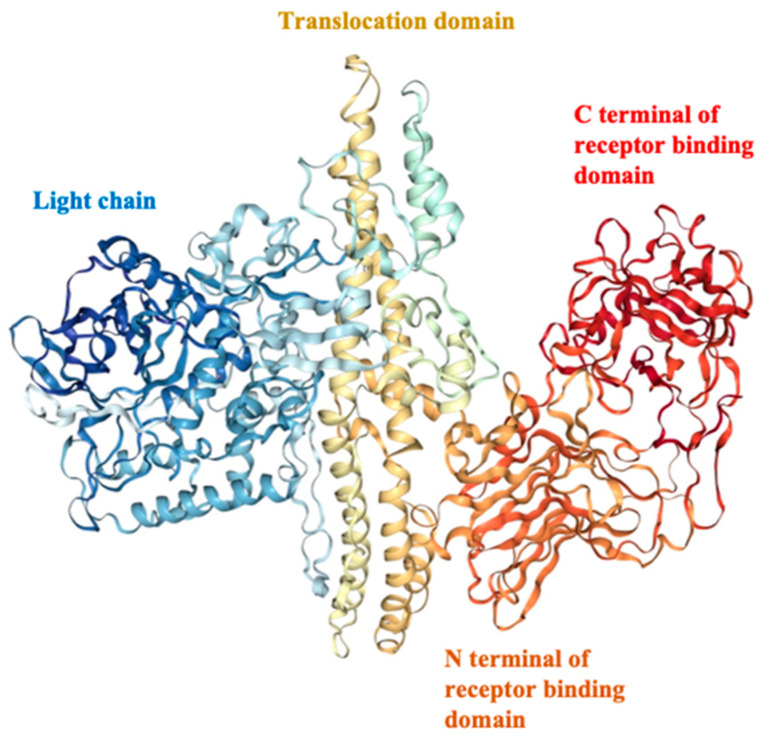
Crystal structure of botulinum neurotoxin (3BTA): crystal structure of botulinum neurotoxin serotype A. The image was obtained from the Protein Data Bank (PDB), ID 10.2210/pdb3BTA/pdb [63].

BoNTs (botulinum neurotoxins) are the most potent protein toxins known to humans to date [58]. There are seven immunologically distinct BoNT serotypes (A–G), with natural variants within each serotype termed subtypes [59]. BoNTs share overall structural similarity with TeNT. They also are 150 kDa protein toxins that are proteolytically processed to form a 100 kDa heavy chain (HC) and a 50 kDa light chain (LC) linked via a disulfide bond (Figure 4). Unlike TeNT, which is produced as a single 150 kDa polypeptide, BoNTs are produced by their clostridial hosts in complex with several non-toxic proteins (NTNH (nontoxic non-hemagglutinin (NTNH)) and HA (hemagglutinin) or orfx proteins). The LCs are zinc proteases that specifically cleave plasma membrane or vesicle-associated SNARE (soluble N-ethylmaleimide-sensitive factor activating protein receptor) proteins [60]. The HC can further be functionally and structurally divided into an H_N_ domain (translocation domain) aiding the LC in entering the cell cytosol and an H_C_ domain (Receptor-binding domain) that specifically associates with ganglioside and protein receptors on neuronal cells [61,62].

BoNTs primarily target motor neurons, and unlike TeNT, the BoNT LCs are released directly into the cytosol of the motor neurons, where they cleave SNAREs at the neuromuscular junction, resulting in inhibition of neurotransmitter release by the motorneurons. This leads to long-lasting flaccid paralysis. Once inside a neuron, BoNT LC can stay active for a prolonged time of days to several months depending on the BoNT serotype [64,65,66]. A vaccine to BoNTs, thus, has to be highly efficient in removing the toxins from the circulation before neuronal cell entry. The major five kinds of botulism are infant botulism, wound botulism, foodborne botulism, iatrogenic botulism, and very rarely, adult intestinal toxemia [67,68]. Wound botulism can occur when spores of botulinum toxin-producing clostridia enter a deep wound and produce the toxin during growth within the wound, similar to tetanus. Foodborne botulism occurs after consuming botulinum neurotoxin already present in improperly canned, preserved, or fermented homemade foods, as well as occasionally from store-bought foods. Infant botulism typically occurs when infants consume contaminated substances, such as honey or soil, consisting of *Clostridium botulinum* spores. The bacteria further grow and produces toxin in the baby’s intestines. Since an infant’s digestive system is not fully developed, they are more susceptible to the bacteria’s growth and toxin production. Iatrogenic botulism refers to botulism that is caused by medical or healthcare interventions. It is a rare form of botulism that can occur when botulinum toxin is administered for therapeutic or cosmetic purposes but is not properly administered, monitored, or controlled.

No cure exists for botulism, but protective vaccination with botulinum neurotoxin toxoid, prepared by formalin inactivation similar to TeNT and DT toxoids, has been shown to be effective in preventing botulism [69,70]. While vaccinating the general population against BoNTs is not desirable due to the significant clinical benefits provided by pharmaceutical BoNTs, certain populations are more vulnerable to the disease and would benefit from vaccination. Since 1965, the CDC has made an investigational pentavalent BoNT toxoid (PBT) vaccine available to workers at risk of exposure under an investigational new drug (IND) application (BB-IND 161), including laboratory workers and some military personnel [16]. The primary vaccination series included injections at 0, 2, and 12 weeks, followed by annual boosters. Moderate and severe local reactions were reported in 10% and 3% of vaccine recipients after the boosters, respectively, while <0.3% reported systemic reactions (inflammation, itching along with nausea, vomiting, or diarrhea), among the approximately 4200 individuals who received the vaccine [71]. Prior to the mid-1990s, around 50% of vaccine recipients had anti-BoNT antibody levels of 0.2 International Units (IU)/mL or higher at their 2-year post-booster assessment. However, by 2004, less than 15% of vaccine recipients had the same levels of antibodies and there was a decrease in the production of antibodies against toxin serotypes C, D, and E, indicating waning vaccine efficacy. Also, potency studies on vaccine lots manufactured in 1976 and distributed since 1982 showed that they no longer effectively protected guinea pigs from serotypes D and E [62]. Eventually, due to declining potency and efficacy and the high prevalence of local side effects, the pentavalent (ABCDE) botulinum toxoid vaccine was discontinued in 2011 [71,72,73]. No human vaccine for BoNTs has been approved since. However, there have been multiple efforts for the development of new vaccines, including recombinant protein vaccines against parts of the BoNT toxins or mutated holotoxins with diminished biologic activity [74,75,76,77,78]. Today, there remains a significant requirement for the development of an effective vaccine for BoNTs.

While in the US the main focus for new BoNT vaccine development is using novel approaches of genetically de-toxified or biologically inactive protein subunit vaccines, scientists in Japan developed another tetravalent chemically detoxified vaccine within the last two decades [79]. This vaccine encompasses formalin-inactivated toxoids of BoNT serotype /A, /B, /E, and /F, utilizing the progenitor M toxin complexes (BoNT and NTNH) [70]. A small human clinical study (*n* = 43) showed neutralizing antibodies against all four BoNTs, with antibody titers increasing with each boost and then slowly waning over time [79,80,81]. While it is unclear why the study used the M-complex including NTNH for vaccine development, this small clinical study looks promising, with 86% of participants reaching protective neutralizing antibody levels above 0.25 IU/mL [70]. In another study in Japan, BoNT/A toxoid combined with a mutated cholera toxin produced BoNT-specific IgG in plasma and IgA in external secretions (saliva, fecal extracts, and nasal washes) in mice [80]. Mice receiving this nasal vaccine were completely protected against challenge with 4000 mouse lethal dose 50 (LD50) Units of BoNT/A by challenge through the intraperitoneal route and against 2 LD50 Units of oral delivered progenitor BoNT/A complex [80]. Taken together the studies on BoNT toxoid vaccines show that chemical detoxification of BoNTs can produce an efficient vaccine that elicits a strong neutralizing humoral immune response to the toxins. However, data on immune memory are limited and yearly boosters were required for most vaccine recipients of the investigational pentavalent BoNT toxoid vaccine to maintain neutralizing antibody levels. Another consideration for BoNT toxoid vaccines is the significant challenges associated with large-scale active toxin production. In contrast to tetanus toxin, against which most people are vaccinated, there currently is no vaccine against BoNTs, and thus the large-scale production of active BoNTs presents a significant biosafety concern for involved laboratory staff. In addition, in the USA, BoNTs are Tier 1 Select Agents, and thus production also involves significant regulatory hurdles associated with high costs. Therefore, newer developments focusing on alternative approaches that do not require large-scale production of the toxins would be of value.

## 5. Recombinant Subunit and Toxin Vaccines

**Figure 5:**
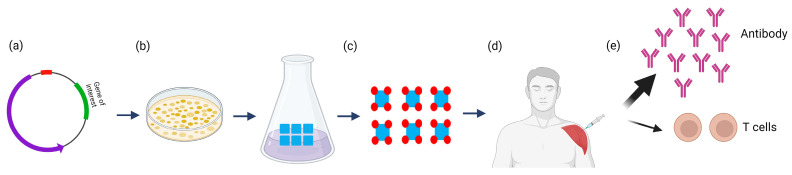
Development of recombinant subunit vaccines: (**a**) Plasmid encoding immunogenic genes, (**b**) Plasmid is transformed into expression host cells (bacteria, yeast or animal cells), and antigen is expressed and purified. (**c**) The purified antigenic protein is formulated with adjuvant to boost immune response. (**d**) The vaccine candidate is injected into the host body. (**e**) The injected antigen triggers a host immune response, resulting in generation of antibodies specific to the antigen. Image created with BioRender.com (accessed on 1 August 2023).

In the last decades, our understanding of molecular mechanisms underlying pathogenesis, as well as genetic methods and protein engineering technology, have advanced dramatically. This enabled the design of recombinant subunit vaccines consisting of specific surface antigens of the pathogens (Figure 5). This approach has the advantage that specific immune-dominant or biologically essential antigens can be used to elicit a strong immune response for effective neutralization and immune clearance of the pathogen. In addition, these vaccines do not involve whole organisms and, thus, completely overcome safety concerns due to potential strain reversion or ineffective pathogen killing [82,83]. On the other hand, subunit vaccines target only the specific protein or subunit provided and lack the myriad of additional epitopes that can be found on whole organisms. They also lack the capacity to invade host cells as the attenuated or killed pathogen vaccines often do, thereby restricting the T-cell immune response. In general, proteins alone do not elicit strong enough immune responses for vaccination; thus, they are formulated with adjuvants to stimulate the immune system. Alum (aluminum salts) is an economical and safe adjuvant approved by the United States Food and Drug Administration (US FDA) for veterinary and human use and is commonly used for protein subunit vaccines. Alum works by forming a short-term depot at the site of injection and slowly releasing antigens to the body’s immune response system [84,85]. Additional adjuvants with specific immunomodulatory functions are actively investigated and hold great potential to improve protein subunit and recombinant toxin vaccines by modulating the induced immune response [86,87,88].

Recombinant subunit vaccines are known to elicit strong humoral and CD4+ T cell responses [8,9], along with poor induction of cellular immune responses, particularly CD8+ T cells [8,9]. Therefore, multiple boosters and adjuvants are required to induce immune memory for long duration [89,90,91]. One well-known example of a recombinant protein vaccine currently used in humans is the vaccine against the hepatitis B virus (HBV). Hepatitis B is a global liver disease, which can result in serious life-long chronic illness. The first HBV vaccine was produced in 1981 and was a killed vaccine derived from the plasma of asymptomatic carriers [92]. Despite a very good safety profile, the high cost of this vaccine combined with the theoretical risk of contamination with other blood-borne viruses such as human immunodeficiency virus (HIV), resulted in the gradual replacement of this vaccine with a recombinant subunit vaccine starting in 1986 [92]. To produce the recombinant vaccine, the hepatitis B surface antigen (HBsAg) is expressed in yeast cells in the absence of any other viral DNA. The resulting recombinant B surface antigen is capable of assembly into virus-like particles (VLPs) of similar size as the virus, which are equally immunogenic and effective as the plasma vaccine [93,94]. Another example of a recombinant vaccine is the vaccine against human papillomaviruses (HPVs), which cause various mucocutaneous diseases, including cervical, vulvar, and vaginal cancers, as well as genital warts [9].

The same vaccine strategy that is applied for recombinant subunit vaccines is also proving valuable in the design of a new type of toxin vaccine, where the toxin is genetically inactivated by the introduction of specific amino acid mutations that render the toxin a biological null. The only currently used example of a recombinant genetically inactivated exotoxin is CRM197. CRM-197 is a genetically detoxified form of the diphtheria toxin (63,000 Da) that is widely used as a carrier protein of conjugated vaccines. To create CRM197, a single mutation at position 52, substituting glutamic acid for glycine, causes the ADP-ribosyltransferase activity of the native toxin to be reduced by about 1 million fold [95,96]. As a result, CRM197 is no longer capable of causing the severe illness associated with diphtheria [95]. However, it retains the ability to stimulate an immune response when used as a component in vaccines and has found great utility as a carrier protein for polysaccharides or small peptides that alone would not be able to induce an immune response, due to its availability of multiple conjugation sites. Examples are the conjugate vaccines to protect against Haemophilus *influenzae* type b or *Streptococcus pneumoniae*. CRM197 has also been conjugated to epidermal growth factor (EGF) to create the EGF-CRM197 vaccine as a potential anti-tumor vaccine for cancers that secrete high levels of EGF. These results are important for future vaccine research and understanding the mechanism of CRM197’s anti-tumor effect [97]. Why has CRM197 not replaced the current diphtheria toxoid vaccine? In addition to production cost, caution is warranted when using this recombinant protein at a high dosage due to studies indicating remaining residual toxicity and weak catalytic activity in cultured cells, although >1 million fold lower than the wild-type diphtheria toxin [98]. The human LD50 for DT (diphtheria toxin) has been estimated at 100 ng/kg; thus, for CRM197, one could extrapolate an LD50 would be 100 mg/kg. The dose required to induce immunity is likely >10,000–100,000-fold lower than this estimated LD50. Further research is required to determine the safe dose effectiveness of CRM197 as a protective vaccine compared to diphtheria toxoid. The following sections will discuss similar developments with tetanus and botulinum neurotoxin vaccines.

### 5.1. Recombinant Tetanus Toxin Vaccines

The current chemically inactivated tetanus toxoid vaccine has been successfully used since 1924 to prevent tetanus, reducing cases by 95%, and continues to be widely used. However, there are several disadvantages associated with this commonly used vaccine. The primary disadvantage is that it requires large-scale production and purification of the highly potent tetanus toxin in its active form before it is inactivated. Even though laboratory workers can be immunized with the current vaccine, reducing the biosafety risk, the large amounts of toxin handled nevertheless present a safety and security risk, and regulatory approvals are required. Only a few manufacturers currently are willing and approved to produce TeNT at the large scale needed for vaccine production, and vaccine shortages occur periodically. Other disadvantages of the current toxoid vaccine include a relatively high incidence of local reactions in 25–85% of patients and severe allergic reactions in ~1 in 100,000 vaccine recipients [35,99,100]. In addition, an initial series of three shots must be followed by periodic boosters about every ten years to maintain immunity [99]. While some improvements to the current tetanus toxoid vaccine may be possible by adjustments in the formaldehyde inactivation process, removal of trace amounts of formaldehyde in the final vaccine, as well as adjuvant formulation, exciting new developments are investigating genetically inactivated recombinant toxin vaccines as an alternative. Research studies have explored atoxic toxin subunit domains, such as the recombinant non-tagged Hc domain of the tetanus toxin expressed in *Escherichia coli* as a recombinant TeNT subunit vaccine [101]. Another recent study developed a genetically detoxified version of tetanus toxin called 8MTT, which retained 99.4% similarity to the original toxin but had eight amino acid mutations to disable several of the intoxication steps of TeNT, including neuronal cell association, translocation of the LC into the cell cytosol, and enzymatic activity of the LC [15]. The modified toxin was purified and after injection into outbred mice at 0.6 mg 8MTT/mice, no symptoms or pathology were observed, suggesting that *E.coli* produced 8MTT is at least 50 million-fold less toxic than native tetanus toxin, which had an LD50 of ~6 pg/mouse in the same study [15]. Vaccination of mice with 8MTT resulted in a strong and robust immune response, which was similar to the immune response in mice vaccinated with an equal dose of chemically inactivated tetanus toxoid after one boost but showed a more pronounced immune response and enhanced survival to toxin challenge after two boosts [15]. In addition, a recent study suggests that 8MTT could also serve as a conjugate vaccine carrier for a small peptide vaccine (tick peptide P0) [102]. Since this peptide is too small to mount an immune response by itself, conjugation to a carrier protein is required. Comparing the immune response in mice immunized with the 8MTT-P0 conjugate versus the chemical TeNT toxoid-P0 conjugate demonstrated an at least equal and strong immune response, validating the 8MTT as a conjugate vaccine carrier. Thus, 8MTT offers an alternative to tetanus toxoid as a primary vaccine and as a conjugate vaccine carrier that is safer to produce, is chemically well defined, and does not require extensive formaldehyde inactivation. Additional studies are needed to further confirm the safety and effectiveness of this approach. Of particular interest would be a combined CRM197—8MTT vaccine.

### 5.2. Recombinant Botulinum Neurotoxin Vaccines

Similar to the development of recombinant TeNT vaccines, this approach is also investigated for the development of recombinant-engineered BoNT vaccines [69]. However, in the case of BoNTs, vaccine development is complicated by the immunogenic diversity of the BoNT serotypes, in effect requiring the development of a multivalent vaccine protecting against all BoNT sero- and subtypes that can cause human botulism. The previously available investigational pentavalent BoNT toxoid vaccine had many downsides associated with it, including the need to produce large amounts of active toxins, reactions to residual formaldehyde in the formulation, varying efficacy for the five included serotypes, the need for yearly boosters, and overall waning efficacy. This eventually led to its discontinuation in 2011 [71], and no approved vaccine has been available since. In addition to serving as a vaccine for at-risk populations, this vaccine also enabled the development and production of Human Botulism Immune Globulin Intravenous (BIG-IV, BabyBIG^®^), which is derived from donated plasma of vaccinated individuals and enables the safe treatment of infants suffering from infant botulism, to both milden disease severity and reduce disease duration [103]. Discontinuation of the vaccine thereby also resulted in a stop in the supply of plasma for further production of this important drug. Thus, efforts to produce new anti-BoNT vaccines commenced rapidly, and in an effort to avoid the downsides previously encountered with the toxoid vaccine, the new efforts focused on recombinant vaccine developments. Recombinant engineered HC, LCH_N_, and full-length BoNT expressed in the yeast *Picchia* or *Escherichia coli* have been studied as vaccine candidates in animal studies. BoNT/A1-H_C_ expressed in *E. coli* had been shown to elicit an antibody immune response against H_C_, resulting in partial protection from challenge by 1200 LD50 units of BoNT/A [104]. Considering that BoNTs require a functional H_C_ domain to associate with neuronal cells for cell entry, this approach targeted the most obvious domain of BoNTs that would result in toxin neutralization. Further studies examined additional HC domain constructs produced in yeast or *E. coli* and found consistent protection against botulism in vaccinated mice [105,106,107]. The H_C_ of all seven BoNT serotypes (hepta- H_C_) has been successfully expressed in *E. coli*. When outbred mice were vaccinated with hepta-H_C_, they developed antibodies against all seven BoNT H_C_s and were protected from challenge by 10,000 LD50 Units of each BoNT serotype [108]. In a small human clinical study, a recombinant H_C_ A and B vaccine produced in the yeast *Picchia pastoris* was assessed in a small cohort of human volunteers who had previously been immunized with the pentavalent toxoid vaccine [109]. The study resulted in a satisfactory 4-fold increase in neutralizing antibodies against BoNT/A and BoNT/B in 82 and 87% of study participants, and several participants donated their plasma for continued production of BIG-IV [109].

The H_C_ vaccines are easy to produce and provide protection against intoxication. However, studies have shown that the LC and H_N_ domains of BoNTs are more immunogenic than the HC domains [66,110], suggesting that optimal vaccines should include these domains. Other studies have demonstrated that even if the H_C_ is modified to lack ganglioside binding, it can still stimulate protective immunity in outbred mice [105,106,107,111], as well as that catalytic activity of the BoNT/LC can be diminished by introducing two to three single amino acid mutations that prevent coordination of the zinc in the LC [74,112,113]. This led to the idea of genetically inactivated holotoxin vaccines that are not able to produce toxicity but maintain their overall structure and immunogenicity. Multiple such BoNT holotoxin constructs with reduced toxicity have been designed and produced in various expression hosts including *Clostridium* [114], *E. coli* [115,116], *Picchia pastoris* [117], and insect cells [118]. All these constructs utilized two to three single amino acid mutations within the LC domain of BoNT to render the toxin catalytically inactive by preventing the coordination of the zinc within the endopeptidase. The toxicity of these constructs was dramatically reduced compared to wild-type BoNTs, ranging from detected residual toxicity of 56 µg/kg to 5 mg/kg in mice, compared to ~150 pg/kg for native toxins [118]. However, as these constructs were produced in heterologous hosts and usually with protein tags to facilitate purification, at least some of the reduction in toxicity may be due to a lack of or sub-optimal post-translational proteolytic processing of the toxin or to protein tags. Since proteolytic processing theoretically can still occur in vitro, including after injection into a host due to host enzymes or by production in a different expression host, further research of these mutant proteins produced in a native expression host is required to directly determine the effect of the individual amino acid substitutions on BoNT toxicity potential.

In a recent study, structure-function knowledge of BoNTs was employed to create holotoxin constructs with distinct amino acid mutations designed to eliminate several biologic functions of BoNT including cell association and LC endopeptidase activity [77,106,119]. The resulting 4M-BoNT/A (mutations) produced in *E. coli* resulted in no detectable toxicity at doses 1 million fold greater than the lethal dose of BoNT/A [120]. However, it is still unclear whether this reduction in toxicity is due to the four introduced single amino acid changes or due to factors related to *E. coli* production and purification.

Several studies evaluated BoNT holotoxin constructs as genetically inactivated recombinant vaccines, comparing vaccine efficacy to toxin subunit vaccines including the H_C_ domains or LC-H_N_ domains [117]. In all studies, the full-length genetically inactivated BoNT vaccines resulted in the greatest and quickest production of protective antibodies and the most protection against the BoNT challenge, including multiple subtypes of each serotype. Interestingly, the LC-H_N_ vaccines resulted in almost equal protection against the BoNT challenge and only slightly reduced overall IgG production [121]. In addition, analyses of the antibodies formed in mice vaccinated with full-length BoNT vaccines showed that the majority of antibodies recognized the LC and H_N_ domains [122]. This is also in agreement with studies analyzing human sera from botulism patients, which indicated that the LC and H_N_ domains of BoNTs appeared the most immunogenic [123,124,125]. However, since antibodies against the H_C_ domain would be expected to mask receptor binding and thereby neutralize toxin cell entry, the inclusion of the H_C_ domain into vaccine design might still play an important role. In addition, immune memory has not been analyzed with these vaccines yet and remains an outstanding challenge [74,107,120,126,127,128,129,130]. In terms of production, the H_C_ and LC-H_N_ domains are easily produced in *E. coli* and production can be scaled to high levels [107]. Production of stable and soluble holotoxin, on the other hand, has proven challenging, and further work is required to optimize such production. It should also be noted that the LC-H_N_ constructs have the potential to enter neurons at very high concentrations; thus, the catalytic activity of a LC-H_N_ vaccine should be eliminated.

Overall, the approach of a recombinant genetically inactivated protein toxin vaccine is promising. Further work is required to both, strengthen our understanding of the molecular mechanisms and structure-function relationships of BoNTs, and to determine mutations resulting in complete elimination of the biologic activities of BoNTs while not altering the overall structure for optimal antigenic epitope display. In addition, direct comparisons of recombinant toxin vaccines with corresponding chemical toxoid vaccines in terms of safety, efficacy, and ease of production will determine the practical utility of this approach. The idea of preserving overall protein structure by avoiding chemical treatments is targeted at creating a vaccine with greater efficacy. However, even if vaccine efficacy is equal to that of the toxoid vaccine, as has been observed for the 8MTT vaccine [15], the advantages of producing an atoxic protein versus a highly potent and regulated neurotoxin are significant from a safety and financial perspective.

### 5.3. Recombinant Subunit Vaccines to SARS-CoV-2

The global COVID-19 pandemic that started in 2019 required rapid development of an effective vaccine. The coronavirus strain responsible for COVID-19 is severe acute respiratory syndrome coronavirus 2 (SARS-CoV-2) [131,132]. SARS-CoV-2 belongs to the Betacoronavirus genus of the *Coronaviridae* family and shares close genomic similarities with SARS-CoV, an earlier endemic virus that first emerged in 2002–2003. SARS-CoV-2 and SARS-CoV are both positive-sense single-stranded RNA viruses with a genome size of ~30 kilobases that encode several structural and non-structural proteins. Scientists around the globe worked quickly to determine the structure, RNA sequence, and pathogenesis mechanisms of SARS-CoV-2 [133,134,135,136,137]. While during the pandemic, vaccine development focused on the production of mRNA vaccines encoding the binding domain of the S protein, recombinant protein vaccines have also been a popular subject of research [138]. The structural proteins of SARS-CoV-2 contain the spike (S) glycoprotein, envelope (E) protein, membrane (M) protein, and nucleocapsid (N) protein [139,140,141]. Since the spike proteins from SARS-CoV-2 (S) are crucial in virus attachment and entry into host cells via interaction with the angiotensin-converting enzyme 2 (ACE2) receptor, S proteins emerged as a key target for vaccine development to induce neutralizing antibodies [142]. Subunit vaccines exclusively focus on targeting a specific protein or subunit, disregarding the multitude of other epitopes present in complete organisms. For example, analyses of COVID-19 patient sera showed antibodies to over 800 distinct immunogenic epitopes on the SARS-CoV-2 virus, with only a subset of these present on the spike protein that was used in the widely distributed mRNA or viral vector vaccines [143,144]. Since the spike protein is essential for cellular entry by the virus and also plentifully adorns the surface of the SARS-CoV-2 virus, the specific targeting of the multiple antigenic epitopes present in the spike protein-binding domain provided a strong neutralizing antibody response protecting from severe disease [145]. The spike protein, however, is also a frequently mutated protein of this virus, thus requiring constant updating of the vaccine.

A vaccine candidate based on the SARS-CoV-2 virus spike protein named “NVX-CoV2373” was developed and produced by biotechnology company, Novavax, based in the United States. Novavax had reported positive results from Phase 3 clinical trials conducted in the United Kingdom [146]. This protein subunit vaccine is shown to be capable of providing immunity against SARS-CoV-2 infection [147,148,149]. However, since the S protein retains its biologic property of being able to bind to the ACE2 receptor, certain sub-populations may be susceptible to deleterious side effects, as has also been observed with the corresponding mRNA vaccine [147,150]. An interesting approach to decrease such a risk of side effects would be introducing specific amino acid mutations that prevent ACE2 binding but maintain overall structure, similar to the approach used in the design of genetically inactivated protein toxin vaccines.

## 6. mRNA Vaccines

The concept of mRNA vaccines was first introduced in the early 1990s, but technological hurdles and delivery challenges delayed significant progress until recent years. Prior to the COVID-19 pandemic, several mRNA vaccines were already in clinical trials to treat various infectious diseases, including HIV-1, rabies, Zika, influenza, and multiple hematologic and solid organ malignancies [151,152,153]. During the COVID-19 pandemic, mRNA vaccine technology rose to prominence, and the most widely used early vaccines against SARS-CoV-2 were based on mRNA vaccine technology.

**Figure 6:**
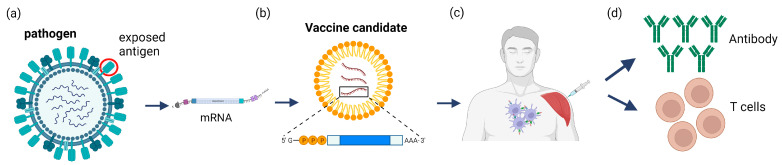
An overview of development strategy for mRNA vaccines. (**a**) The gentic sequence from the pathogen antigen codes for synthetic mRNA sequence. (**b**) Synthetic mRNA is packaged into lipid nanoparticle. (**c**) The vaccine is injected into the host. The mRNA is delivered to immune cells, resulting in production of antigen. (**d**) The vaccine stimulates host’s immune response resulting in production of antibodies and T cells specific to the antigen. Image created with BioRender.com (accessed on 1 August 2023).

The mRNA vaccines are based on mRNA encoding a bacterial or viral protein that is immunogenic enough to elicit an efficient humoral or cellular immune response in the host body upon administration. The mRNA is packaged within nanoparticles for cellular delivery and is modified such that it can evade the host cell’s immune defenses. Once inside the cells, mRNA gets translated by host cell ribosomes to create a protein antigen. In most cases, the protein antigen contains a secretion peptide sequence for efficient release from the host cell into the patient’s circulatory system, where the host’s immune response mounts an effect leading to an immune response [154] (Figure 6). The mRNA vaccines directly deliver mRNA to the cytoplasm, bypassing the nucleus and avoiding incorporation into the genome. Its presence in cells is short-lived, and it is quickly broken down and eliminated through cellular processes. The mRNA vaccines can be designed, formulated, and mass-produced very rapidly, unlike traditional vaccines that take months to develop [152,153,154]. The ability to rapidly produce large amounts of vaccine was of great importance during the COVID-19 pandemic. A disadvantage of mRNA vaccines, however, is that they need to be stored at very cold temperatures (−80 °C), requiring logistics and costs associated with a cold storage chain for global distribution. Another disadvantage of these vaccines was the relatively new technology of nanoparticle delivery and the relative lack of safety data. However, after the COVID-19 pandemic, millions of mRNA vaccines have been administered to patients demonstrating a very low incidence of severe side effects and no deaths due to the vaccine.

### 6.1. SARS-CoV-2 mRNA Vaccines

Due to the swift worldwide transmission of SARS-CoV-2 in the initial year of the COVID-19 outbreak, the mRNA vaccine approach was adopted as a primary method for expeditious and efficient vaccine production. The vaccines were based on synthetic mRNA strands encoding the SARS-CoV-2 spike glycoprotein, packaged in lipid nanoparticles.

Of the multiple recombinant vaccines approved in the United States against COVID-19, including Pfizer-BioNTech, Moderna, Novavax and Johnson & Johnson’s Janssen, the two most frequently used and earliest available were mRNA vaccines. BNT 162b2 (Pfizer) and mRNA-1273 (Moderna) were approved for emergency use by the Food and Drug Administration (FDA) in less than a year [155,156]. Both vaccines were delivered intramuscularly with at least two doses required for protection, and a third dose booster for longer-lasting immunity [155,156]. These vaccines were the first widely used human mRNA vaccines after they were authorized for emergency use in multiple countries around the world. While there was significant debate on the idea of widely distributing these vaccines without the usual safety studies required in vaccine developments, we have now gained great insight into the safety and efficacy of mRNA vaccines after 2 years of widespread applications and over 5.5 billion people, or 72% of the entire world population, having been vaccinated against COVID-19. The side effects associated with these vaccines included pain, tenderness, swelling, or redness at the injection site along with fatigue, headache, muscle pain, chills, joint pain, fever, nausea, malaise, and lymphadenopathy [155,156]. According to clinical trial data and real-world studies, the reported incidence of lymphadenopathy following COVID-19 vaccination varies between 0.3% to 16% of vaccinated individuals. These numbers can fluctuate depending on factors such as the vaccine used and the specific population studied. [157]. In rare cases, a severe allergic reaction occurred within one hour of receiving either vaccine [158,159]. Another serious side effect that has been linked to mRNA-based COVID vaccines is an increased risk of temporary and treatable myocarditis or pericarditis in up to 154 per one million vaccine recipients, particularly in young male populations [160]. However, the risk of developing myocarditis or pericarditis due to COVID-19 infection is about 6 times higher than the risk of developing this condition from the vaccine [161]. Further, Johnson & Johnson’s Janssen (J&J/Janssen) (Ad26.COV2.S) vaccine, which was the third most commonly used vaccine early on in the pandemic, utilized an adenoviral vector to encode and display the SARS-CoV-2 S protein on a replication-deficient virus particle, a technology that has previously been used for the Ebola Virus Vaccine [162], the Human Immunodeficiency Virus (HIV) Vaccine, and the Malaria Vaccine [163,164]. Similarly, the University of Oxford/AstraZeneca (AZD1222(ChAdOx1_nCoV19) COVID-19 vaccine used an adenovirus vector producing the SARS-CoV-2 S protein [165]. However, these vaccines were associated with a risk of thrombocytopenia syndrome (TTS) resulting in blood clots. After a total of ~14 million people received the J&J vaccine in the US, and the Oxford/AstraZeneca vaccine underwent phase 1, 2, and 3 clinical trials, TTS was reported in 3.23 per million doses of J&J vaccine administered and in 7.5 per million doses of Oxford/AstraZeneca vaccine administered, with ~20–40% of TTS cases leading to death [166]. While the incidence of TTS was very rare, particularly compared to the case fatality rate of COVID-19 (0.1–5% depending on country), the cause underlying the development of TTS remains unknown and distribution of this type of vaccine in the US was halted in 2023. The mRNA vaccines, on the other hand, had a high incidence of mild side effects and a low incidence of more serious but temporary and treatable side effects. Importantly, no deaths have been attributed to the mRNA COVID vaccines. In addition, Guillaine–Barre syndrome, which is a rare auto-immune condition that sometimes develops after any vaccinations, was found to be 21 time lower after mRNA vaccinations compared to adenoviral vector vaccines against COVID-19 [32]. Recent studies have begun to investigate the molecular mechanisms underlying myocarditis pathogenesis after the SARS-CoV-2 S protein mRNA vaccines and found no difference in immune response but an increase in circulating full-length spike protein, indicating the possibility that the spike protein may have a biologic effect [167,168]. Further research elucidating the pathogenesis mechanism may lead to our ability to design mutated biologically inactive spike protein antigens, either as protein vaccines or as mRNA vaccines.

### 6.2. Developments in mRNA Immunotherapy to Botulinum Neurotoxins

Even before the mRNA vaccines developed during the COVID-19 pandemic, this technology was examined as a countermeasure for botulism using a passive vaccine strategy. Two elegant studies have shown that mRNA vaccines encoding antibodies or antibody fragments can be used to protect against botulism after toxin exposure. In this strategy, host cells take up the nanoparticle-coated mRNAs and antibody fragments are rapidly produced by the host cells and released into the circulatory system to neutralize botulinum neurotoxins before neuronal cell entry [169,170]. One of the studies explored the use of chemically unmodified mRNA to produce neutralizing antibody derivatives [169]. This study designed mRNA encoding heavy chain only VH-based neutralizing agents (VNAs). VNAs have significant biophysical advantages compared to traditional antibodies due to the small size of the VH-domains (14 kDa), ease of production, improved stability, and tissue penetration characteristics. A single injection of mRNA-lipid nanoparticle (LNP) resulted in rapid and long-lasting serum VNA titers, detectable 2 h post injection, providing prophylactic and therapeutic protection against botulinum toxin intoxication [169]. Overall, this study demonstrated the effectiveness of an mRNA vaccine as a potent technology for passive immunization. In a second study, the same group employed mRNA encoding linked VH-domains to create VNAs that recognize multiple serotypes of BoNTs [170]. This study created multiple heterohexameric VNA antitoxins composed of six camelid sdAb (single domain antibody) components (VHs), with two capable of neutralizing BoNT serotypes, A, B, or E, respectively. To test the potential of mRNA therapeutics encoding long sdAb heteromultimers, a heterohexamer was encoded as replicating RNA (repRNA), formulated with a cationic nanocarrier, and delivered to mice via intramuscular injection. Heterohexamer antitoxin serum expression levels were detectable by 8 h post treatment, peaked at 5–10 nM around two days, and remained for more than three days [170]. The heterohexamer constructs protected mice from at least 100 MIPLD50, (mouse intraperitoneal median lethal dose) of each serotype [170]. This study demonstrated the potential of long sdAb multimers, which can be administered as proteins or repRNA, in the development of antibody-based therapeutics [170].

Overall, these developments indicate a promising approach that may be extended to protective passive vaccination strategies for other toxins or organisms in the future, in particular, if safety barriers based on protein studies are included to ensure expressed antigens will be atoxic in the host. The studies described here used knowledge gained from monoclonal antibody (mAb) studies to select optimal neutralizing antibodies [171]. The mAbs-based therapies are expanding, but mAB production is expensive and not easily scaled to high output levels. Utilizing this mRNA approach provides a less expensive and easy-to-scale alternative, although there is a delay of several hours between the administration of the mRNA and the production of the neutralizing antibodies. Can a similar mRNA approach be used to create a protective anti-BoNT vaccine by encoding the toxin? In addition to hurdles due to the large size of the protein toxin, which would require the construction of a large mRNA, the idea of using mRNA to produce toxins inside the host cells is negated by the toxicity of BoNTs. However, biologically inactive subunits or genetically inactivated holotoxins may be good candidates for such developments. Detailed structure-function studies, toxicity analyses, and pharmacokinetics on the protein level will be necessary to ensure the safety and efficacy of such an approach.

## 7. Discussion and Future Directions

Vaccines have been one of the greatest contributors to improved population health. The recurring question of whether vaccines are safe for use often arises, in particular when new technologies are introduced. While vaccine safety varies based on the vaccine technology used as well as the pathogen, our medical history has shown that vaccines are always safer than the diseases the vaccines are meant to prevent or lessen in severity, and there has been a steady progression in increased safety over time.

Whole organism vaccines have the theoretical and, in some cases, demonstrated the possibility of the vaccine strain reverting to a pathogenic strain or being incompletely inactivated [22]. Recombinant protein or subunit vaccines, on the other hand, completely eliminate this risk as they do not contain whole organisms and rely on host immune responses against identified immunodominant antigens from bacteria, viruses, or toxins [8]. Also, since these vaccine formulations consist of recombinant protein specifically, protective immunity against infection is achieved without potential risks associated with other parts of the pathogen [9,172]. Recombinant and toxoid vaccines are comparatively favorable in terms of production, transportation, and storage. The vaccine candidate can be lyophilized to powder form, has a better shelf life, and can be transported and stored at room temperature as well [173]. On the other hand, recombinant and toxoid vaccines are more costly to develop and usually require the addition of adjuvant as part of vaccine formulation to achieve effective immune responses, and inducing a protective T-cell response and immune memory in addition to the humoral response is challenging. Recombinant protein vaccines can be replaced by viral vector vaccines, which in general have been clinically successful [174]; however, questions such as the use of viral vector backbone and its impact on immune response, in particular with boosters vaccinations, remain unanswered [175,176]. Viral vector vaccines are inappropriate for toxin vaccines, as they would result in the production of toxins in the host and the potential incorporation of toxin-encoding DNA into the host genome. Utilizing specific mutations in the toxin gene to knock out toxicity, as is currently being explored for recombinant toxoid vaccines, would mitigate the risk of active toxin production, but still, a risk of DNA incorporation and reversion remains. This risk is mitigated by utilizing an mRNA approach, which results in a short burst of (mutated and inactivated) protein expression followed by the destruction of the mRNA.

Interestingly, both mRNA and its specific delivery systems provide a self-adjuvanting effect resulting in long-lived and robust adaptive immune responses in mRNA-based vaccines [177]. As mRNA theoretically can encode any antigen, they can be designed against numerous targets and toxin variants with minimal formulation and adaptation to antigen alteration [178], with rapid manufacture. However, mRNA vaccines require specialized storage and transport conditions, including ultra-cold temperatures, which can be challenging and expensive. This characteristic becomes much more significant when the vaccine technology is required to be transferred to low- and middle-income countries, where limited resources are available to manufacture and store mRNA-based vaccines (storage and transportation of the vaccine vials at (−70 °C/−94 °F) are not easily accessible) [179,180,181].

All recombinant and toxoid vaccines share the necessity of booster shots at regular intervals to generate a strong and effective immune response, and particularly to create immune memory [18]. Table 1 provides an overview of the vaccine technologies discussed in this manuscript and their advantages and disadvantages.

Although there have been remarkable advancements in vaccine development in recent years, it is important to recognize that not all vaccines can generate a defensive immune response in the host or confer lifelong protection to all recipients. To improve existing vaccines, new strategies are required to address unmet needs, particularly for pathogens with antigenic hypervariability (such as human immunodeficiency virus) or pathogens with multiple serotypes such as dengue viruses and *S. pneumoniae*, and for pathogenic exotoxins [9]. Advances in areas such as recombinant DNA technology, bioinformatics, molecular biology, and immunology have provided helpful insights into understanding the interaction of pathogen cells and toxins with host cells. These advancements have the potential to lead to the development of multiple new vaccine strategies by specifically targeting subunits or toxin domains involved in host pathogenesis. However, in conjunction with discovering safe and effective antigens, adjuvants, and delivery systems, the challenges of vaccine development extend to evaluating the balance between cost, benefits, and risks before translating a vaccine candidate to the clinic. The success of vaccines in the future relies on determining which technology, alone or in combination, is optimal for each pathogen or toxin. This answer again is dependent on the pathogen or toxin properties and mechanisms leading to pathogenesis in the host. Overall, much work remains to be carried out at the forefront of vaccine developments, to optimize vaccine candidates, production strategies, and immune-modulating adjuvants. Much of what is learned from vaccines targeting whole pathogens or subunits can be applied to the design of improved toxin vaccines, and visa versa. For botulinum and tetanus toxin vaccines, recent research determined that the entire toxin protein results in the most robust immune response, and that it is possible to introduce relatively few targeted amino acid substitutions to delete the biologic activity of the toxin while maintaining overall structure and antigenic epitopes. The same idea of selectively mutating single amino acid residues to create a vaccine consisting of a biologically null recombinant protein can also be utilized to improve the safety profile of recombinant subunit vaccines, some of which may result in undesired side effects in specific subpopulations. Finally, this approach is also easily translated towards mRNA vaccines, where the mRNA code is easily altered to produce a biologically inactive but highly immunogenic protein. Most promising perhaps is the combination of toxicity research using recombinant toxins and subunit proteins, and the mRNA technology for vaccine delivery. Overall, recent progress in vaccine technology and our understanding of molecular mechanisms involved in pathogenesis offer a promising and bright future for new vaccine developments.

## Figures and Tables

**Table 1:** Advantages and disadvantages corresponding to different vaccine types.

Vaccine Type	Advantages	Disadvantages
Live vaccines (attenuated)	▪ Elicit humoral as well as cellular immune responses. ▪ Long-lasting protection ▪ Single dose is normally sufficient.	▪ Risk of reversion to pathogenic strain. ▪ Complex attenuation process required. ▪ Vaccine composition not well defined.
Killed vaccines	▪ The vaccine strain does not revert back to pathogenic strain. ▪ Usually only mild side effects such as fever and nausea observed. ▪ Inexpensive to produce at mass level.	▪ Multiple boosters required. ▪ Addition of adjuvants required. ▪ Vaccine elicits primarily humoral responses. ▪ Vaccine composition not well defined. ▪ Large amounts of live pathogens must be cultured to produce vaccine antigens.
Toxoids	▪ Toxicity inactivated. ▪ Low incidence of mild adverse events such as soreness or swelling, fever, nausea. ▪ Good stability. ▪ Doesn’t require cold storage.	▪ Multiple doses required to achieve immune memory. ▪ Addition of adjuvants required. ▪ Local reactions to residual formaldehyde used for inactivation. ▪ Requires large-scale production of active toxins. ▪ Immune epitopes after inactivation procedure not well defined.
Subunit vaccines	▪ Composition well defined. ▪ Can be formulated with various vaccine delivery systems such as adjuvant, viral vector, nanoparticles, etc. ▪ Live pathogen is not used to produce the antigen. ▪ The vaccine strain cannot revert back to pathogenic strain.	▪ Multiple doses or boosters required for immune memory. ▪ Antibody response restricted to subunit present in vaccine. ▪ Addition of adjuvants required. ▪ Live pathogens may be cultured to purify the vaccine antigen.
Recombinant toxin vaccines	▪ Can utilize atoxic toxin sub-domains. ▪ Can inactivate toxicity by specific mutations without altering immunogenic surface epitopes. ▪ Safe to produce as no active toxin is involved.	▪ Multiple doses or boosters required. ▪ Addition of adjuvants required. ▪ More expensive and complex to develop compared to other vaccine types.
mRNA vaccines	▪ Can be quickly designed, tested, and mass produced. ▪ Do not consist of live pathogens. ▪ Large number of uses during COVID pandemic indicates good safety and effectiveness.	▪ Requires storage in extreme cold conditions. ▪ Multiple doses or boosters required. ▪ More data on safety are required. ▪ Potential of adverse side effects caused by produced antigen.

## Data Availability

Not applicable.
